# A Trusted Routing Scheme Using Blockchain and Reinforcement Learning for Wireless Sensor Networks

**DOI:** 10.3390/s19040970

**Published:** 2019-02-25

**Authors:** Jidian Yang, Shiwen He, Yang Xu, Linweiya Chen, Ju Ren

**Affiliations:** 1 School of Software, Central South University, Changsha 410083, China; gideonyang@csu.edu.cn (J.Y.); chenlwy@csu.edu.cn (L.C.); 2 School of Computer Science and Engineering, Central South University, Changsha 410083, China; xuyangcsu@csu.edu.cn (Y.X.); renju@csu.edu.cn (J.R.)

**Keywords:** wireless sensor networks, trust, routing scheme, blockchain, reinforcement learning, delay performance, efficiency

## Abstract

A trusted routing scheme is very important to ensure the routing security and efficiency of wireless sensor networks (WSNs). There are a lot of studies on improving the trustworthiness between routing nodes, using cryptographic systems, trust management, or centralized routing decisions, etc. However, most of the routing schemes are difficult to achieve in actual situations as it is difficult to dynamically identify the untrusted behaviors of routing nodes. Meanwhile, there is still no effective way to prevent malicious node attacks. In view of these problems, this paper proposes a trusted routing scheme using blockchain and reinforcement learning to improve the routing security and efficiency for WSNs. The feasible routing scheme is given for obtaining routing information of routing nodes on the blockchain, which makes the routing information traceable and impossible to tamper with. The reinforcement learning model is used to help routing nodes dynamically select more trusted and efficient routing links. From the experimental results, we can find that even in the routing environment with 50% malicious nodes, our routing scheme still has a good delay performance compared with other routing algorithms. The performance indicators such as energy consumption and throughput also show that our scheme is feasible and effective.

## 1. Introduction

Wireless sensor network (WSN) is a promising technology to collect and send information to the clients through the self-organization network in the way of a single-hop or multi-hop relay, which has a wide application prospect in military national defense, environmental science, industry, agricultural automation and other fields [1,2,3,4,5]. WSN is composed of a large number of micro-integrated sensor nodes, which work together to complete environmental monitoring, environmental perception and collection of various information. The multi-hop routing technology is one of the key technologies of WSN and is mainly responsible for transmitting the data information collected by sensor nodes from source node to destination node according to the agreed routing protocol [6]. However, the open, distributed and dynamic characteristics of WSN make the multi-hop routing vulnerable to various types of attacks, thus seriously affecting the security and effectiveness [7,8,9]. Traditional secure routing schemes are targeted at the specific malicious or selfish attacks and are not suitable for multi-hop distributed WSN as they mainly rely on the encryption algorithm and authentication mechanism.

**Figure 1:**
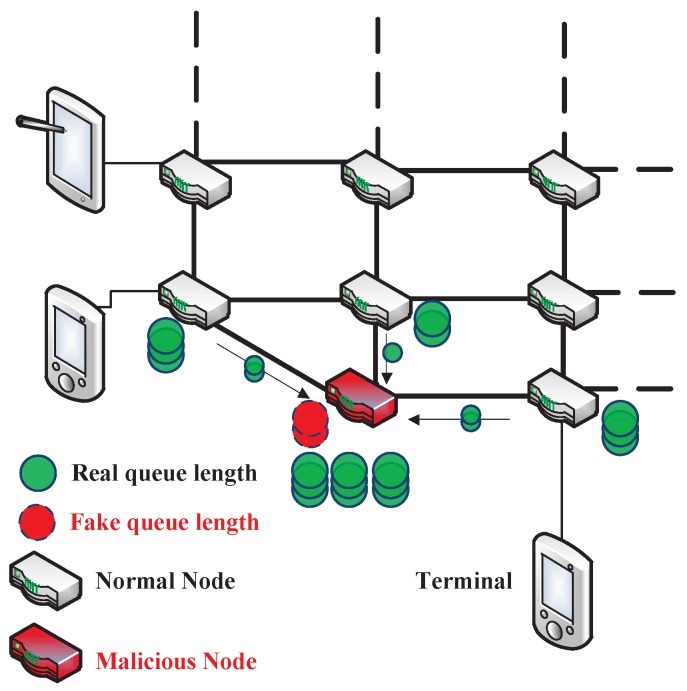
The malicious node in a backpressure (BP) routing algorithm.

In time-varying and dynamical WSN environments, existing routing schemes cannot accurately distinguish the malicious nodes. In some specific routing algorithms, the routing nodes cannot distinguish the truth of routing information released by other routing nodes. As shown in Figure 1, a malicious node can release a false queue length information to increase the probability of receiving packets, thus affecting the routing scheduling of other routing nodes [10]. The existing routing schemes find it difficult to identify such malicious nodes, because the real-time change of the routing information between two routing nodes are difficult to be accurately distinguished. When a malicious node receives the packets of data from a neighbor node, it directly discard the packets and does not forward the packets of data to its next-hop neighbor node. This creates a data “black hole” in the network, hence it is named as a black hole attack which is hard to be perceived for routing nodes in WSNs [11,12]. These malicious nodes may be attackers of the external intrusion or internal legitimate nodes captured by external attackers. Recently, trust management is a pervasive means to ensure the security of the routing network [13,14,15,16,17,18,19]. Its core approach is for each node to maintain and exploit a trust model that records the trust values of the neighbor routing nodes and make routing decisions. This method can effectively make the routing node choose the relatively reliable routing links according to the trust values. However, its application is limited since a single routing node can only get the trust values of the neighbor routing nodes, which is not completely compatible with the multi-hop distributed WSN.

In view of the above security issues, a third-party intermediary is proposed to solve the trust problem between the routing nodes, but the intermediary is obviously not suitable for multi-hop distributed wireless sensor networks. Meanwhile, a third-party trust management center is likely to be attacked and controlled by malicious nodes, and therefore the security and fairness of the system cannot be guaranteed. As a trusted, decentralized, self-organizing ledger system, the blockchain is very suitable for multi-hop distributed wireless sensor networks [20,21,22,23]. A lot researches on applying the blockchain to the routing algorithms are carried out in the past few years [24,25,26]. The blockchain is essentially a decentralized database maintained by multiple nodes, and it mainly deals with trust and security problems. We’ve summarized four core technical elements that enable the blockchain to provide trusted and secure services:

(i) The first is the distributed ledger which contains all the transactions on the blockchain. The contents of these transactions include the address of the receiver of the transaction, the amount of the transfer, the timestamp, the smart contract code, the execution result of the smart contract, etc. The transaction ledger is completed jointly by multiple nodes in different places. Each node in the blockchain keeps a complete ledger, so that no ledger information can be tampered. While all of the nodes can participate in monitoring the legality of transactions.
(ii) The second is the asymmetric encryption and authorization technology. The transaction information stored on the blockchain is public, but the account identity information is highly encrypted and can only be accessed under the authorization of the data owner, thus ensuring the security of data and personal privacy.
(iii) The third part, called consensus mechanism, is how all accounting nodes reach consensus to determine the effectiveness of a blockchain transaction, which is a means of preventing tampering. Some common consensus algorithms including proof of work (PoW), proof of stake (PoS), proof of authority (PoA), delegated proof of stake (DPoS) and proof of capacity (PoC) are discussed in [27,28]. We introduce three mainstream blockchain consensus mechanisms relevant to our work:
  - PoW: Bitcoin, Dogecoin and Litecoin are among the digital currencies based on the PoW consensus mechanism. PoW algorithm relies on the node to carry out mathematical operations to find a random number and obtain the accounting right. A malicious node needs more than 51 percent of the network’s computing power to take control of the blockchain network. Compared with other consensus mechanisms, the resource consumption of the PoW blockchain is high and the supervision is weak. At the same time, every time a PoW consensus is reached, the whole network needs to participate in the operation, which has low performance and efficiency.
  - PoS: The main idea of the PoS consensus mechanism is that the difficulty of obtaining the accounting right of a node is inversely proportional to the stake held by the node. According to the proportion and time of coins taken by each node, the difficulty of mining coins can be reduced in the same proportion so as to speed up the speed of finding random numbers. The greater the stake, the greater the privilege, the greater the responsibility to generate the block and the power to generate more revenue.
  - PoA: PoA is an improved algorithm of PoS that uses the verified identity of the nodes to replace the role of the stake rather than the monetary value. In a PoA blockchain, the transaction and the block are validated by an approved node (called a validator) without a huge computational overhead of a mining process. The validator must authenticate on the blockchain and the qualification is hard to acquire which means the validator will not have a motive for acting on their own interests. Even if there is a malicious validator, it will be kicked out by other validators’ votes. In this way, the PoA blockchain becomes safer and cheaper.
(iv) The last technical element is the smart contract, which is based on the trusted and non-tampering data and can automatically execute the predefined codes by a blockchain miner [29]. The execution result of the smart contract updates the ledger status on the blockchain network. These changes cannot be falsified or tampered with once they are confirmed by a specific consensus mechanism because the content has been agreed upon in the blockchain network.

In an open, reliable and distributed blockchain network, a routing node can acquire routing information including but not limited to its neighbor routing nodes. The efficiency of the routing can be improved if this routing information is properly used. Some routing schemes have introduced the reinforcement learning into the dynamically networks [30,31,32,33,34]. Reinforcement learning is a kind of machine learning algorithms represented by Q-learning, which gives the feedbacks into the selection of each step through the reward and punishment mechanism. A standard reinforcement learning algorithm consists of five parts: environment, agent, state, action and reward [35]. An agent interacts with the environment by performing actions. A state is an indicator of the situation of the agent, and each state has a corresponding set of actions for the agent to choose. The agent can only perform one action per state and get the reward which is the feedback on the success or failure of the action.

In this work, we introduce a novel trusted routing scheme based on blockchain and reinforcement learning for WSNs. In particular, we use the blockchain technology to provide a distributed routing information management platform that all the routing information is recorded on the blockchain through the blockchain token transactions. The scheme takes advantage of the decentralized, tamper-proof and traceable characteristics of the blockchain transactions to improve the trustworthiness of the routing information between the routing nodes. We exploit the reinforcement learning to learn the dynamic, reliable and extensive routing information from the blockchain network. A dynamically updated reinforcement learning model is generated in each routing node through the dynamically updated reward value brought by the action (scheduling) of each state (packet location), so as to help the routing nodes make better routing decisions and select the more reliable and efficient routing links.

## 2. Related Work

In this part, we firstly review the traditional trusted routing schemes for improving the routing security and reliability. Then, we introduce the relevant research approaches of routing schemes using the blockchain technology. Finally, we investigate the state-of-the-art of the application of the reinforcement learning in routing networks.

### 2.1. Traditional Trusted Routing Schemes

Providing a trusted routing environment is an important and difficult issue for WSNs. There are many related researches to implement a trusted routing scheme. Li et al. designed a novel trust-based routing protocol by extending the widely used AODV (ad hoc on-demand distance vector) routing protocol [15]. The protocol applies a trust model to recommend the trusted routing nodes and improve the security of the routing environment. Later, there are more researches on a trust-based routing scheme. In [16], Lu et al. proposed a secure routing scheme by quantifying and recording the algorithm-compliance behaviors of the routing nodes. Sirisala et al. proposed a QoS (quality of service) routing algorithm to evaluate the trustworthiness of the routing nodes [17]. The algorithm calculates the direct QoS trust of the 1-hop neighbor routing node, the indirect trust of the 2-hop neighbor routing node is calculated by the transitive rule (e.g., A trusts B and B trusts C then A trusts C). Some researchers embed the trust mechanism into the routing paths [18,19], so that the trusted routing paths were scheduled. Most of these researches are based on a “reputation system”, which evaluates the reputation of other nodes to make routing selection. However, building a reputation table requires the historical behaviors of the routing nodes which cannot guarantee the real-time security of WSNs. Meanwhile, the reputation table maintained by each routing node may be tampered with, so that the absolute credibility cannot be guaranteed.

### 2.2. Blockchain-Based Routing Schemes

Recently, some people combined the tamper-proof and traceable characteristics of the blockchain technology with routing algorithms to improve the trustworthiness between the routing nodes. Gómez-Arevalillo et al. presented a trusted public key management framework named secure blockchain trust management (SBTM) [24]. The approach replaces the traditional public key infrastructure (PKI) with a blockchain protocol, thereby removing the central authentication and providing a decentralized inter-domain routing system. In [25], Li et al. established a multi-link concurrent communication scheme based on the blockchain technology. According to the specific integrated factor communication tree (IFT) algorithm and the behavioral characteristics of the routing nodes in the blockchain-based communication, the nodes can be classified as malicious or non-malicious. Ramezan et al. proposed a blockchain-based contractual routing (BCR) protocol for routing networks with untrusted nodes [26]. It utilizes smart contracts to help routing nodes find a trusted route to the destination nodes. The main principle is that the source node confirms the routing arrival of each hop on the smart contract and records the malicious routing nodes with malicious behaviors. The subsequent packets will then no longer pass through a known malicious node. However, the scheme has security risks that a malicious node with the BCR tokens can falsely claim to have received the packets.

### 2.3. Reinforcement Learning Algorithms in Routing Schemes

**Figure 2:**
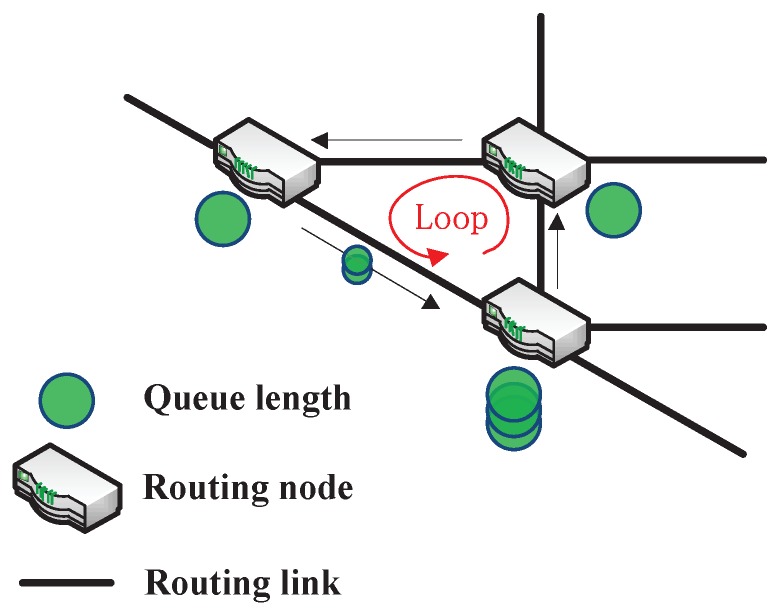
Loop routing problem in routing algorithms.

It is difficult to effectively utilize the dynamic routing information in WSN routing networks. A self-adaptive routing algorithm is needed for such dynamic routing networks. To enhance the self-adaptability of routing scheme, Boyan et al. were the first time to combine Q-learning algorithm with packet routing to dynamically learn the routing situation to find the shortest path [30]. Reinforcement learning is a useful tool for mining complex, dynamically updated routing network information to optimize the routing scheduling algorithms. In the traditional backpressure routing algorithm, due to the limited routing information of neighbor routing nodes, the loop routing problem is caused as shown in Figure 2 that results in a huge delay in the whole routing process. Recently, Gao et al. proposed multi-agent Q-learning (QL) aided backpressure routing algorithm named QL-backpressure (BP), where each routing node only needs the local information of the neighbor routing nodes to solve this problem [34]. Their algorithm not only outperforms the BPmin algorithm in delay performance but also contains the excellent characteristics: distributed implementation, low computational complexity, and high-throughput [36]. However, when the malicious nodes appear, the throughput-optimality characteristic will no longer exist. The routing scheme based on reinforcement learning should ensure both efficiency and security. Mayadunna et al. proposed a malicious routing node detection scheme based on the reinforcement learning [37]. The core of the algorithm is to judge whether a node is malicious by dynamically learning the number of packets received by the node’s neighbor nodes. But this solution can only be used to identify black hole attack nodes, which is very limited in the complicated and variable WSN routing environment.

## 3. Approach

In this section, we introduce a novel trusted routing scheme based on the blockchain and reinforcement learning. First, we put forward the threat model of our scheme and briefly describe the attack and cheating methods of malicious nodes in the routing environment. Then, we propose a blockchain-based network architecture to strengthen the credibility of routing information. We also design a specific routing scheduling algorithm based on reinforcement learning for the designed blockchain-based network architecture, labeled as RLBC (reinforcement learning and blockchain based) routing algorithm, in which the reinforcement learning is used to help the routing node select the next optimal routing node. Finally, we analyze the security of the proposed trusted routing scheme.

### 3.1. Threat Model

In this paper, we assume that the blockchain network is trusted, that is, no attacker can control the blockchain network by controlling more than half of the server nodes. We further assume that the routing nodes are untrusted and the vulnerable routing nodes may be controlled by malicious attackers. In a routing scheduling process, a malicious routing node can falsely claim to have sent a certain number of packets to a routing node or deny receiving packets sent by other routing nodes. The malicious routing nodes can release false routing information on the routing network, such as queue length information, thus affecting the routing scheduling process. They can also act as black hole attack nodes and refuse to forward packets. However, we do not consider the collusion attack by two routing nodes to complete invalid blockchain transactions. We further assume that a routing node can only act as a normal or malicious node, which means attacks are by no means intermittent. Meanwhile, we do not consider the occasional abnormal behavior caused by the performance of the node (e.g., a node does not send a message in time or loses the wireless spectrum).

### 3.2. Blockchain-Based Network Architecture

**Figure 3:**
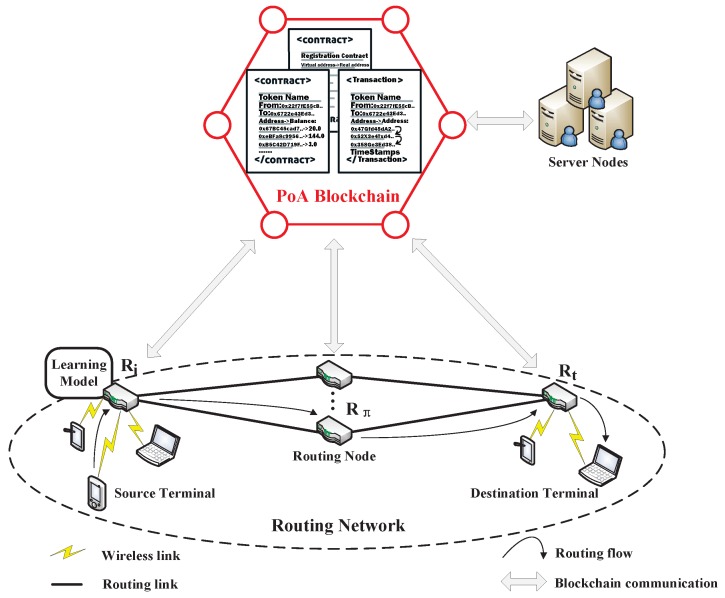
The framework of the blockchain-based routing scheme.

To enhance the trustworthiness and robustness of the routing information, we introduce the blockchain which is essentially a distributed ledger with tamper-proof, decentralization, and information traceability characteristics into the wireless sensor network and use the blockchain token transactions to record related information of each node, as shown in Figure 3. The main framework is divided into two parts: the actual routing network and the blockchain network. In the framework, there are three kinds of entities: server node *S*, routing node *R*, and terminal device.

In Figure 3, the actual routing network consists of routing nodes and terminals. Each routing node *R* has its own LAN to connect several terminals and is responsible for receiving packets from other routing nodes or terminals, as well as forwarding the received packets to the target nodes. We briefly describe the routing flow; packets from the source terminal to the destination terminal are transmitted to a routing node $R_{i}$. $R_{i}$ then selects the next-hop routing node $R_{\pi }$ via the routing policy π obtained by the local learning model. The local learning model constantly queries and collects the relevant routing network state information from the blockchain network. After continuous transmission, the packets will be delivered to the target routing node $R_{t}$ then to the destination terminal.

Each blockchain system has a specific consensus algorithm to ensure the fairness of the blockchain transaction. In our blockchain network, we choose the PoA consensus algorithm which can process transactions more efficiently. In Figure 3, the red lines represent the PoA blockchain network, which is constituted by server nodes and routing nodes. They respectively represent two kinds of entities of the PoA blockchain network with different identities:

- Validator: The validators are the pre-authenticated nodes of the blockchain, which have advanced authorization and are responsible for the verification work in the PoA blockchain. In our system, each server node is a validator with higher rights in the PoA blockchain and has a unique blockchain address. Their specific tasks include executing smart contracts, verifying blockchain transactions, and releasing blocks on the blockchain. A new validator can be added by the authenticated validators election with more than 50% of the votes. Even if there is a malicious validator, it can only attack one of the contiguous blocks at most, during which time the malicious validator can be kicked out by other validator votes.
- Minion: The minions are less-privileged nodes and cannot perform the verification work as validators in the PoA blockchain. In our system, each routing node is also a minion and has fewer rights in the PoA blockchain and it has a unique blockchain address, too. They can initiate token contracts, trigger some contract functions, and query the transaction information on the blockchain.

In our scheme, these nodes in WSN can be static or dynamic. For example, the server nodes in our solution are often static, while the routing nodes can be dynamic. However, the entry and exit of nodes does not affect our scheme, because the status information of our blockchain-based system is also updated dynamically.

On the blockchain network, we use different blockchain tokens to represent the different packets to be delivered to the target nodes that *n* unit tokens represent *n* unit corresponding packets. The essence of a token is the representation of the digitized information of the corresponding packets stored in the smart contract. The routing nodes can initiate token contracts to generate tokens and map the state information of related packets. They will make token transactions with each other via the token contract to transmit the tokens based on the sent and received packets. According to the consensus mechanism between server nodes, the token transactions cannot be revised arbitrarily by malicious nodes, to some extent, the token accurately represents the packet passed between the routing nodes.

Compared with traditional routing architectures, our system differs in that each routing node is registered on the registration contract after entering the blockchain-based routing network. The routing node will forward the packets to its next-hop routing node off the blockchain. Then they must confirm the routing information on the blockchain including the address of the next-hop routing node, the number of packets sent to the next node, and the timestamp. Then the routing information will be confirmed by the server nodes through the blockchain consensus mechanism and updated on the blockchain. The learning model of each routing node will pull this information from the blockchain and feed back the subsequent routing policy to the routing node. In the following subsections, we will introduce the specific blockchain network implementation and the routing policy generation method in detail.

### 3.3. Blockchain Network Procedure

**Figure 4:**
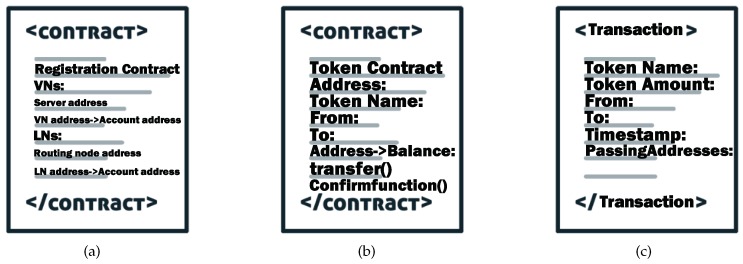
Specific formats of smart contracts and blockchain transactions. (**a**) Registration contract. (**b**) Token contract. (**c**) Token transaction.

To effectively operate the blockchain-based routing network architecture, information related to the routing network needs to be transferred to the blockchain network. The related routing information is recorded in the smart contracts including the registration contract, the token contract, and blockchain transactions, i.e., the token transaction as shown in Figure 4. All of these contents are verified by the server nodes then released to the blockchain.

All the smart contracts are manipulated by the authenticated servers nodes and the results of the execution are returned to the blockchain network. The registration contract records the identity information (e.g., the physical address of a node) of all the routing nodes and server nodes to facilitate the query of the entire network node. The specific procedure for generating the registration contract is described as Algorithm 1. The mapping map is an inherent variable of the registration contract and contains the mapping of the blockchain addresses to the physical addresses. The mapping *state* is also an inherent variable that stores the state of whether a node is registered or not. When a new node wants to register on a registration contract, it should trigger the contract as a contract caller. It then inputs its physical address pa as the identity information, and the registration contract automatically records its blockchain address ba. The registration contract checks whether ba exists in the state mapping or not. If state=0, the registration contract stores the map ba→pa into the map mapping and the map ba→1 into the state array, the result of this operation is a success. If state=1 indicates that the node has been registered, the result of this operation is a failure. The logic of the code makes the blockchain address of the node corresponding to the identity, and the registration information cannot be changed once it is registered.

**Algorithm 1** Procedure of registering a node.
 1: Mapping map: blockchain.address→physical.address;  2: Mapping state: blockchain.address→0 or 1;  3: **while**
true
**do** /* The contract is waiting for a contract caller to trigger */ **Input:** Contract Caller’s Blockchain Address ba; Contract Caller’s Physical Address pa; **Output:** Registration Result *r*;  4:   r←null;  5:  **if**
state(ba) = 1 **then**
 6:     r←failure;  7:  **else**
 8:    map(ba) = pa;  9:    state(ba) = 1;  10:    r←success;  11:  **end if**
 12: **end while**


**Figure 5:**
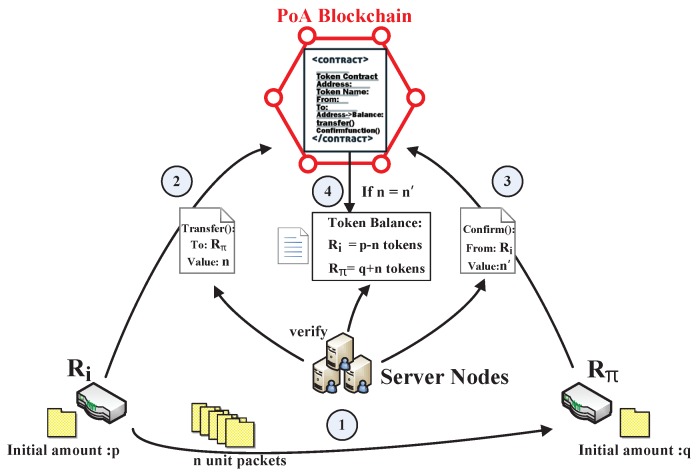
Implementation of the token transaction.

Each routing node can release a token contract to generate a certain number of tokens by giving the correlated variables on the blockchain [29]; the releaser’s blockchain address, the token name, an empty table mapping the routing node *R* to the token balance $B_{R}$, the supply of the tokens, the destination routing node $R_{t}$ of the tokens, etc. After releasing the token contract, the contract will automatically generate a corresponding supply of tokens for the releaser. Then the routing nodes can transact the tokens by triggering the “transfer” and “confirm” functions of the token contract. The process of transferring tokens is recorded as a token transaction on the blockchain. Figure 5 depicts the implementation details of one complete token transaction and we divide a token transaction into three processes:

(i) We initialize the number of packets in $R_{i}$ as *p* and in $R_{\pi }$ as *q*, i.e., $B_{R_{i}}$ = *p* and $B_{R_{\pi }}$ = *q*. First, $R_{i}$ transmits *n* unit data packets to $R_{\pi }$. In the traditional routing network, $R_{\pi }$ will send back an acknowledgement (ACK) to confirm that the packets have been received and the token balances should be $B_{R_{i}}=p-n$ and $B_{R_{\pi }}=q+n$. Meanwhile, each routing node performs routing scheduling based on the routing information released by neighbor routing nodes in the traditional routing network. However, if $R_{\pi }$ is a malicious routing node, it can deny the routing process to its neighbor routing nodes, such that the token balances for its neighbor routing nodes will be $B_{R_{i}}=p$ and $B_{R_{\pi }}=q$, so such these schemes cannot guarantee the trustworthiness between the routing nodes.
(ii) In our scheme, we put the process of validating the routing process on the blockchain network and all the routing nodes get the relevant routing information from the blockchain network instead of their neighbor routing nodes. After the packets are transmitted, $R_{i}$ trigger the “transfer” function on the token contract to indicate the state change including the information of the amount *n* of tokens sent to $R_{\pi }$ to the blockchain network. Then the token amount *n* is released on the token contract. While $R_{\pi }$ trigger the “confirm” function on the token contract to confirm the amount $n^{\prime }$ of the received packets to the blockchain network. The number $n^{\prime }$ is based on the amount of the packets $R_{\pi }$ actually received in the routing network.
(iii) Then the token contract checks whether *n* equals $n^{\prime }$, and if $n=n^{\prime }$, the token balances for $R_{i}$ and $R_{\pi }$ end with $B_{R_{i}}=p-n$ and $B_{R_{\pi }}=q+n$. If $n\neq n^{\prime }$, the token balances for $R_{i}$ and $R_{\pi }$ remain $B_{R_{i}}=p$ and $B_{R_{\pi }}=q$. The whole token transaction is confirmed by the PoA consensus of the server nodes, i.e., only the validation of more than half of the authenticated server nodes can allow a server node to upload the transaction to the blockchain network. We stipulated that the whole transaction process should be completed within one time slot. The unconfirmed transactions are cancelled and a failed transaction is not recorded in the blockchain without affecting the routing information.

Meanwhile, every token transaction is recorded on the blockchain by an authenticated server node, that all the routing nodes and server nodes have a back-up of the transaction recorder available for traceability. Each token transaction records the token name, the timestamp $t_{i}$, the amount $n_{i}$ of tokens transmitted and the route addresses $A_{t}$ of each hop arrived. Since all the transaction information is supervised jointly by all network nodes, our blockchain-based platform provides a distributed routing environment, enabling the acquisition of global routing information. With this blockchain-based routing scheduling model, it is possible for routing nodes to apply and obtain the routing information dynamically from the blockchain network. It can also provide a trusted routing environment for traditional routing algorithms (e.g., the backpressure routing algorithm). However, its routing information has not been effectively utilized, and the performance needs to be improved. We introduce the reinforcement learning algorithm to dynamically learn this information, and all of the information will be captured by the reinforcement learning model of the routing node to output subsequent routing policies and help the routing node choose the better routing links.

### 3.4. RLBC Routing Algorithm

In this part, we elaborate the reinforcement learning-based routing scheduling scheme by using the global, dynamic, and trusted routing information provided by the proposed blockchain platform. The information obtained by the learning model from the blockchain network includes the timestamp $t_{i}$ of the token transaction (all transactions represented by array $T_{t}$), the transfer amount $n_{i}$ of tokens for each hop routing node (all amounts represented by array $N_{t}$), the amount of remaining tokens, and the address array $A_{t}$ passed by. The specific reinforcement learning and blockchain based (RLBC) algorithm is summarized in Algorithm 2. Environment *E* represents a real routing environment, where the routing nodes update their states and make decisions about actions. State *x* represents the current position of the packets that need to be transferred, i.e., the packets locate currently at the *x*-th routing node. It means that the learning model has *n* states if there are *n* routing nodes. Policy π(x) is the action *a* to be taken from the current state *x* and the action *a* represents forwarding the packets to the next-hop routing node $R_{a}$. It means that when a state the routing node has *m* next hop nodes to choose from, the current state has *m* actions. Therefore, the action space A(x) represents the collection of all actions at routing node *i*. For example, $R_{1}$ has two and $R_{2}$ has three actions to choose from: A(1) = $\left\{a_{1},a_{2}\right\}$, A(2) = $\left\{a_{1},a_{2},a_{3}\right\}$ (according to the actual situation, the $a_{1}$, $a_{2}$ in the two arrays are not necessarily the same). Note that $q_{t}\left(x,a_{k}\right)$ is the differential queue length between $R_{x}$ and $R_{a_{k}}$ that $q_{t}\left(x,a_{k}\right)$ = $B_{R_{x}}$ − $B_{R_{a_{k}}}$. The size of this metric affects the probability that $R_{x}$ transmits the packets to $R_{a_{k}}$. The larger this metric is, the greater the probability will be. Therefore, if a routing node releases a lower queue length, it can increase the probability that other routing nodes transmit packets to it.

**Algorithm 2** Reinforcement learning and blockchain based (RLBC) routing algorithm.**Input:** Environment *E*; Action Space A; Initial State $x_{0}$; Reward Discount γ; Learning Rate α; **Output:** Policy π;  1: $Q_{t}$(*x*,*a*) = 0, *P*(*x*,*a*) = $\frac{1}{|A(x)|}$;  2: *x* = $x_{0}$;  3: **for**
T=1,2, … **do**
 4:   *a* = $\pi^{p}$(*x*);  5:   *r* = reward by routing action *a*;  6:   $x^{\prime }$ = next state by routing action *a*;  7:   $a^{\prime }$ = π($x^{\prime }$);  8:   $Q_{t}$(*x*,*a*) = $Q_{t}$(*x*,*a*) + α(*r*+$\gamma Q_{t}$($x^{\prime }$,$a^{\prime }$) − $Q_{t}$(*x*,*a*));  9:   π(*x*) = arg $\max_{a_{k}}q_{t}$(*x*,$a_{k}$)$\cdot Q_{t}$(*x*,$a_{k}$);  10:   *x* = $x^{\prime }$;  11: **end for**

In the core Equation (1) at line 8 of Algorithm 2, $Q_{t}$ represents the Q-table of the packets sent to the routing node $R_{t}$. When the packets reach the next routing node $R_{\pi }$ from current routing node $R_{i}$, it enters the next state. The action is moving the packets to the next-hop routing node $R_{\pi }$.

(1) $$Q_{t}\left(x,a\right)=Q_{t}\left(x,a\right)+\alpha \left(r+\gamma Q_{t}\left(x^{\prime },a^{\prime }\right)-Q_{t}\left(x,a\right)\right);$$

Parameter 0≤α≤1 is the learning rate of the Q-learning. Where α equals to one, means that the learning algorithm ignores the initial $Q_{t}$. The reward discount γ represents the specific gravity of the next state. The larger its value is, the greater the influence of the next state on the current $Q_{t}$ will be. The reward value *r* is an important parameter to motivate the routing nodes to make better routing decisions. Its value is determined by the routing information obtained by the learning model from the blockchain. The information includes the timestamp $t_{i}$, the amount $n_{i}$ of transferred tokens, and the address array $A_{t}$. When the routing node $R_{i}$ makes action *a*, the number of nodes and packets it finally reaches is used to measure the reward. In a time slot, the value of *r* is $V_{max}$ when the tokens are successfully delivered to the target node $R_{t}$. When the tokens are delivered to the history node, it means that the address is in $A_{i}$ and the loop routing problem happens, and we set *r* to $V_{min}$. In other cases, the value of *r* is related to the amount of successfully delivered tokens, for example, if the amount of tokens actually transmitted is $n_{i}$, the value of *r* is $n_{i}$. Since the reinforcement learning reward value *r* is determined by the amount of tokens delivered, it also restricts the non-forwarding behavior of malicious nodes in the threat model. If a malicious node doesn’t transmit any packet (token) in the routing process, the value of *r* will be small and it can greatly reduce the possibility that packets will pass through this malicious node. Thus, the problem of blocking routing link caused by malicious black hole nodes is greatly reduced.

By dynamically extracting and learning relevant routing information from the blockchain, the learning model will eventually output a policy π for the routing node. The value π represents the routing policy which determines the next-hop of the packages targeted at $R_{t}$, and it depends on the value of $q_{t}\left(x,a\right)\cdot Q_{t}\left(x,a\right)$. Assuming that, when the state is *x* and the number of *a* in the action space is *K*, the probability distribution of $a_{k}$ is selected based on Boltzmann distribution. The specific equation of Boltzmann distribution is shown in Equation (2). The parameter τ>0 is called “temperature”, and the lower τ>0 is, the higher the probability that the high reward action will be selected, and the packet is passed to the corresponding $R_{\pi }$. In general, the higher the value of $q_{t}\left(x,a_{k}\right)\cdot Q_{t}\left(x,a_{k}\right)$, the more likely it is to execute a policy π(*x*) = $a_{k}$.

(2) $$P\left(x,a_{k}\right)=\frac{e^{\frac{q_{t}\left(x,a_{k}\right)\cdot Q_{t}\left(x,a_{k}\right)}{\tau }}}{\sum_{k=1}^{K}e^{\frac{q_{t}\left(x,a_{k}\right)\cdot Q_{t}\left(x,a_{k}\right)}{\tau }}}.$$

The reinforcement learning based routing algorithm helps the choice of routing by the nodes’ dynamic learning. Each hop of the routing information is recorded on the blockchain, and if the hop is looped, or the link is untrusted, or the transmit rate is low, the algorithm greatly reduces the probability that the packets pass through the link. At the same time, the routing algorithm can dynamically discover the more reliable and efficient routing links, so as to help the routing nodes make better routing decisions.

### 3.5. Security Analysis

We established a trusted routing information management system based on the PoA blockchain, where all the routing nodes and server nodes jointly maintained the routing transaction. In this subsection, we analyze it from six perspectives to show how our proposed scheme ensures the security of the system. The related security performance is shown as follows:

- PoA consensus mechanism: The blockchain network is based on a consensus mechanism called PoA (proof of authority), and only the validation of more than half of the authenticated server nodes can allow a server node to upload the transaction and update the routing information. Therefore, any information on the blockchain cannot be tampered with by individuals.
- Transaction traceability: The server nodes record the transactions on the blockchain, including the transaction of releasing token contract, the transaction of routing node running functions on the contract, the transaction of transferring tokens. All the information about these transactions is recorded on the blocks and can be traceable across the blockchain network.
- Routing information source: Different from the traditional routing network, in our scheme, all the routing nodes get the relevant routing information from the blockchain network instead of its neighbor routing nodes. In this way, the routing information obtained by the whole network routing nodes is consistent and not determined by individuals.
- Avoid the single point attacks: Our blockchain-based routing scheduling scheme does not require a trusted third-party central authority to manage routing information. The single point attack is prevented by the authentication of the transaction by multiple server nodes.
- No double-spending: The codes of our token contract specifies that each routing node address maps to only one address at each time slot, and that the routing node will not initiate token transactions to two other routing nodes at the same time slot.
- Self-adaptability: In the proposed routing scheduling scheme, the routing link with malicious nodes will not generate routing transactions. The reward value *r* of the routing link is very low based on our RLBC routing algorithm and the learning model will adaptively select the routing link of normal nodes.

## 4. Experimental Analysis and Evaluation

To evaluate the effectiveness and performance of our proposed approach, we implemented a prototype and compared its performance of the RLBC algorithm with other routing algorithms. In terms of effectiveness evaluation, it is mainly compared with the state-of-the-art reinforcement learning-based routing algorithm, the trust-based algorithm, and our original blockchain-based algorithm. For performance evaluation, we compared our system with the traditional PoW-based blockchain system to reflect the performance of our system in terms of delay, consumption and throughput.

### 4.1. Testing Setting

We built a PoA consortium blockchain and simulated 32 virtual servers to update blockchain transactions on the chain. All the routing information required by the reinforcement learning model can be obtained from the public blockchain transactions. The consortium blockchain was built based on Geth 1.8.19 which can provide reliable Ethereum transaction services. We chose the BP routing algorithm as the benchmark of performance comparisons [10]. To simulate real packet arrival rates, we simulated 32 terminals in the 16 × 16 matrix, randomly transmitting packets to the target point according to the Poisson distribution with λ packets/slot. We also simulated 16 × 16 routing nodes to receive and deliver real packets in one packet/slot at most based on the routing policy generated by the local reinforcement learning model. The data was finally recorded in the experiment, including average packet delay, transaction delay, energy consumption, etc. The detail configurations of the devices are shown below in Table 1.

To test the effectiveness, we set delay performance experiments in the routing environment with malicious nodes. In order to establish the comparative experiments, we compared our RLBC algorithm with the traditional BP algorithm, the trust-based backpressure (TB-BP) algorithm [18], the state-of-the-art reinforcement learning-based algorithm named QL-BP [34], and our original blockchain-based algorithm. There were 25% and 50% malicious nodes in the 16 × 16 routing nodes to interfere with the normal routing scheduling, that the malicious nodes will try to forge false queue length information and use the vulnerability of BP algorithm to cheat more packets (see in Figure 1) or act as a black hole node and not transmit any packet. Specifically, we assigned three kinds of malicious nodes and they appear with the same probability:

(i) A malicious node releases a fake low (10% of the true amount) queue length information, but it transmits packets to other routing nodes.
(ii) A malicious node releases the true queue length information, but it doesn’t transmit any packet to other routing nodes.
(iii) A malicious node releases a fake low queue length information and it doesn’t transmit any packet to other routing nodes.

To evaluate the effectiveness of our system, we tested the average latency, average energy consumption and throughput of the blockchain token transactions in comparative experiments. We implemented and compared the traditional blockchain system based on PoW consensus mechanism to show the advantages of our system based on PoA consensus mechanism [38].

### 4.2. Effectiveness

We compared the traditional BP algorithm, QL-BP algorithm, and TB-BP algorithm with our system to see whether the routing scheduling schemes can be affected by malicious nodes. We introduced the BP algorithm combined with the blockchain-based (BC) architecture for comparison. The comparative experiments showed the differences between BP, QL-BP, TB-BP, proposed BC and proposed RLBC algorithm in the malicious routing environment. Compared to our system, the other three routing scheduling schemes do not have the addition of a blockchain structure, but the number of malicious nodes and the cheating methods are the same in all of these schemes.

#### Simulation Results

**Figure 6:**
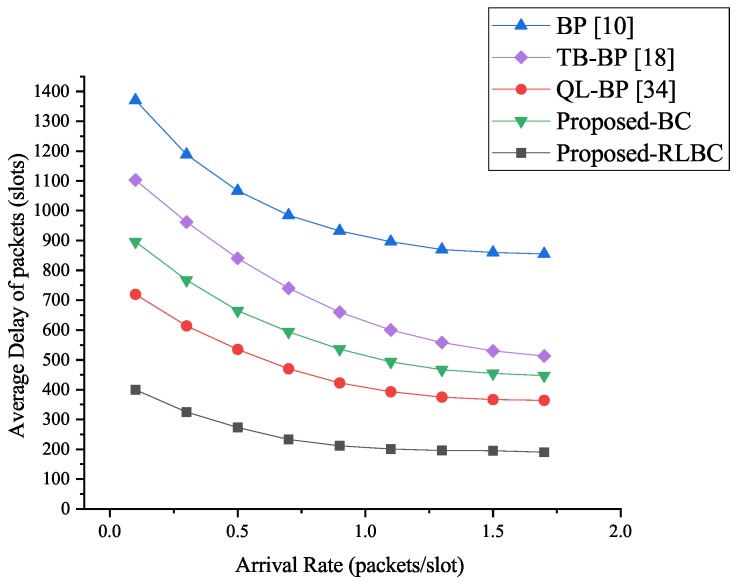
Average delay of packets with 25% malicious nodes.

From Figure 6, we can see that in the routing environment with 25% malicious nodes, the delay performance of our BC algorithm reduces the average packet delay by around 48% when compared to traditional BP algorithm. The performance is similar to that of QL-BP algorithm while the average packet delay reduces about 54%. The packet delay of TB-BP algorithm reduces around 32%. Our RLBC algorithm performs best and it reduces around 78% delay when compared to BP algorithm, 52% compared to the QL-BP algorithm, and 67% compared to the TB-BP algorithm.

**Figure 7:**
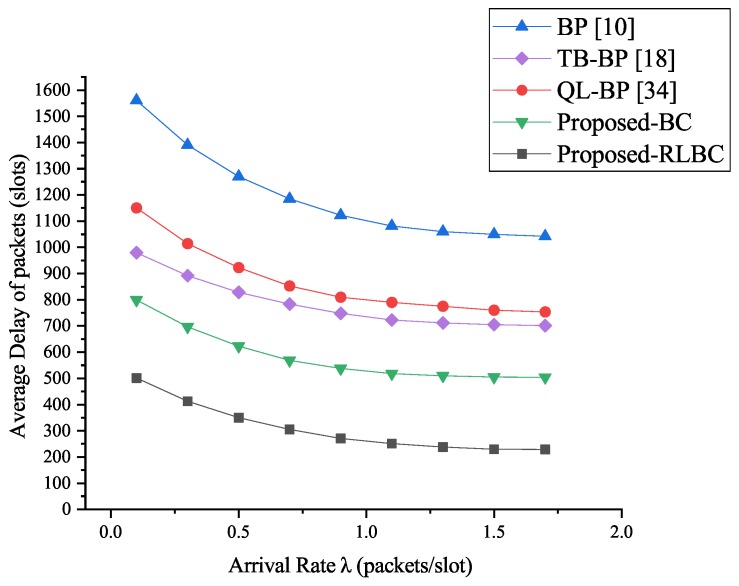
Average delay of packets with 50% malicious nodes.

We also implemented the comparative experiments in the routing environment with 50% malicious routing nodes, and the average packet delay of each algorithm was shown in Figure 7. As we can see, under the influence of a large number of malicious nodes, both algorithms of our system still maintained good performance. Our RLBC algorithm reduced average packet delay by around 81% while the BC algorithm reduced it by around 55% and its performance exceeded the QL-BP algorithm, which it only reduced by around 32%, while the TB-BP algorithm only reduced by around 37%. This was because in QL-BP algorithm, the queue length information released between the routing nodes is not trusted. The malicious nodes can increase the parameter $q_{t}\left(x,a_{k}\right)$ in Algorithm 2 by issuing a false lower queue length, thereby greatly increasing the probability that the data packet is sent to the malicious node and affecting the normal routing scheduling work. The experimental results show that our RLBC algorithm is not susceptible to the influence of malicious nodes in terms of average packet delay, and the effectiveness proved that it is feasible to use it to improve the performance of routing algorithm.

### 4.3. Efficiency

In the efficiency experiment, for a more intuitive comparison, we compared our blockchain system based on the PoA consensus mechanism with the traditional PoW-based blockchain system. We recorded the experimental data such as transaction latency, energy consumption and throughput during the experiment.

#### 4.3.1. Token Transaction Latency

We took the transaction packaging time as the evaluation element of the average token transaction latency, which records the elapsed time that miners put the Ethereum token transaction on the blockchain. We recorded the token transaction latency of PoA and PoW blockchain systems with the increase of arrival rate λ.

**Figure 8:**
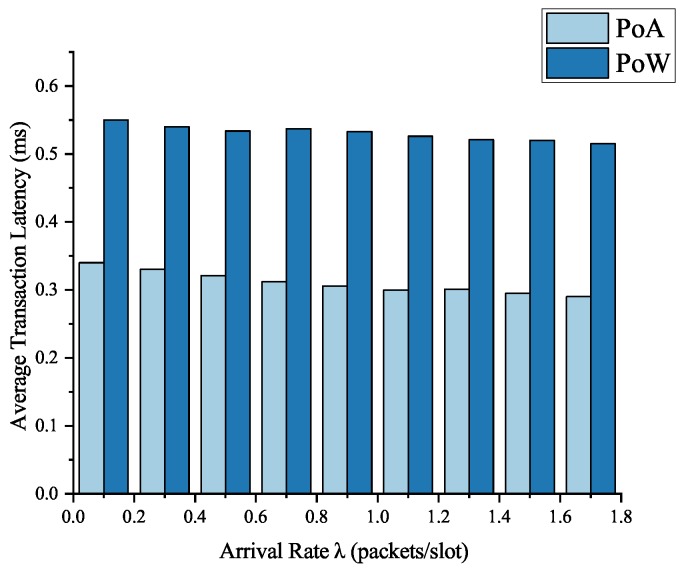
Average transaction latency of proof of authority (PoA) and proof of work (PoW) blockchain systems.

The experiment results are shown in Figure 8, we can see that the latency of the transaction is relatively stable and does not fluctuate much with the arrival rate λ. The average transaction latency of our PoA blockchain system was around 0.29 ms while that of the PoW blockchain system was around 0.52 ms. The results show that our blockchain system based on PoA consensus mechanism can save about 44% of the transaction latency, and obviously, such a token transaction delay is acceptable and has little impact on routing scheduling. It is practical and efficient to use our PoA blockchain system to collect and manage routing scheduling information.

#### 4.3.2. Token Transaction Energy Consumption 

In Ethereum networks, “gas” is a special unit used to measure how much “work” an action or a series of actions of a miner has. It is determined by the number of computer instructions operated by the Ethereum transaction (e.g., the length of the code in the smart contract), and the gas fluctuation does not change much. For example, to calculate a Keccak256 encryption hash value, every time computing the hash will need 30 gas. Ethereum platform trading or contract execution of every operation needs a certain amount of gas, and the more computational resources operation we need, the more gas costs. Then the gas will be converted into corresponding ether currency to pay the blockchain miner. We take the gas consumption as the main evaluation index of the system energy consumption and the experimental results are shown below.

**Figure 9:**
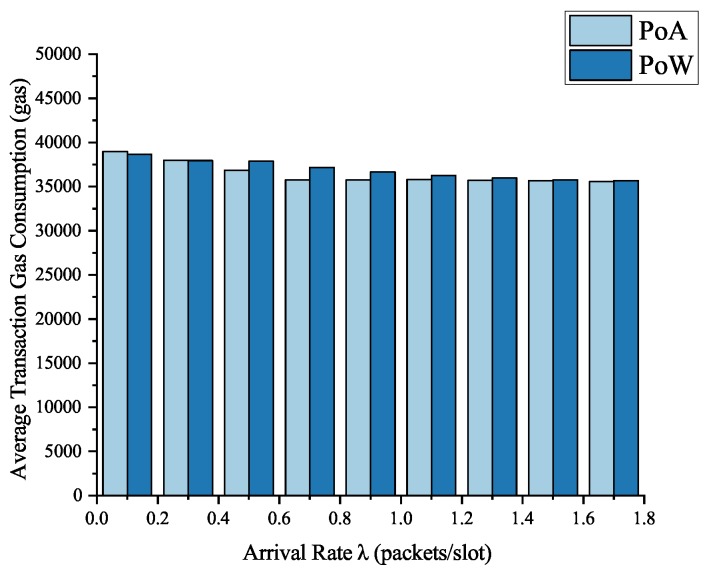
Average transaction consumption of PoA and PoW blockchain systems.

As can be seen from the experimental results in Figure 9, the gas consumption of the average transaction is very stable as the arrival rate λ increases, and since the computing resources required are the same that the consumption of the two systems is approximately equal. With the increase of the arrival rate, the average token transaction gas consumption of the two blockchain systems was finally stable around 35,660 gas which cost only 0.0007132 ether (≈0.0613 USD) at the gas price of 0.02 ether per million gas. In private Ethereum networks, the economic costs will be less and such a consumption tradeoff is small and acceptable.

#### 4.3.3. Token Transaction Throughput

**Figure 10:**
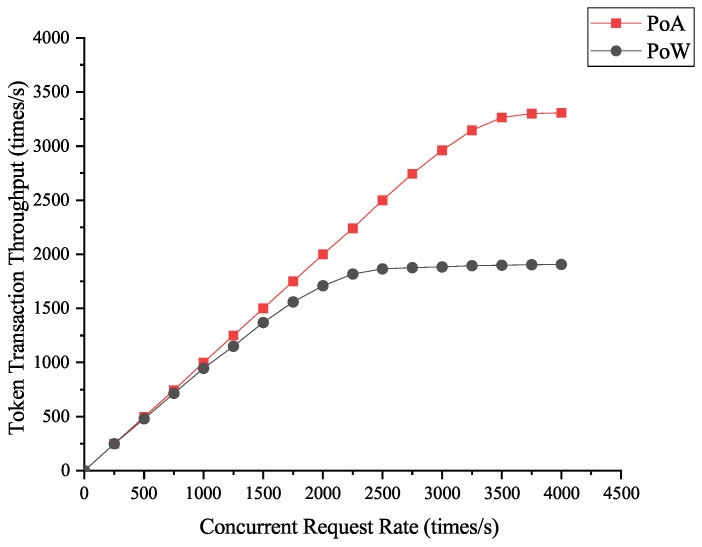
Token transaction throughput of PoA and PoW blockchain systems.

The token transaction throughput shows the blockchain system’s ability to handle concurrent token transactions. We tested the throughput of transactions processed by our PoA blockchain system and the PoW blockchain system under the token transaction request rate ranges from 0 to 5000 times/s. The experimental results are shown in Figure 10.

As we can see, the token transaction throughput increases steadily as the rate of concurrent requests increases, and the curve gradually flattens out as the throughput reaches its peak. Finally, the throughput of our blockchain system based on PoA consensus mechanism is stable at 3300 times per second, while that of the traditional blockchain system based on PoW consensus mechanism is only about 1900 times per second. From the experimental results, we can see that a PoA-based scheme has more efficient transaction processing capacity in the face of high request concurrency. It is appropriate and correct to take PoA algorithm as the consensus mechanism algorithm of the blockchain system, and this PoA blockchain-based routing scheduling scheme can effectively cope with the situation of large concurrent requests in the routing environment.

## 5. Conclusions and Future Work

In this paper, we proposed a trusted routing scheme based on the blockchain and reinforcement learning to provide a trusted routing environment and improve the performance of the routing network. As a decentralized system, the blockchain network provides a feasible scheme for routing information management and a platform for reinforcement learning of routing scheduling. We use the blockchain token to represent the routing packets, and each routing transaction is released to the blockchain network through the confirmation of the validator nodes. By making every routing transaction recorder traceable and tamper-proof, routing nodes can obtain dynamic and trusted routing information on the blockchain network. We also describe the detailed reinforcement learning model to adaptively choose the best routing path and avoid the routing links with malicious nodes. Finally, we carry out simulation experiments, the experimental results show that our system can effectively suppress the attacks of malicious nodes, and the system’s latency and throughput performance are excellent.

In the future, we plan to use our system for experiments in more routing scheduling algorithms besides BP algorithm to verify the effectiveness and portability of our system. We also plan to incorporate the blockchain-based data validation technology into our work [39,40,41,42]. In addition, we intend to put the reinforcement learning model into the blockchain smart contract to simplify the operation complexity and reduce the running burden and expense of the server side. 

## Figures and Tables

**Table 1:** Specifications of devices.

Parameter Name	Server Node	Terminal Node	Routing Node
CPU	2.6 GHz	1.2 GHz	580 MHz
RAM	16 GB	1 GB	32 MB
Storage	1 TB	16 GB	256 MB
Network	1000 Mb	100 Mb	100 Mb
OS	Ubuntu Server 16.04	Raspbian 4.14	OpenWRT 15.05
